# A Single Amino Acid Substitution in the Matrix Protein (M51R) of Vesicular Stomatitis New Jersey Virus Impairs Replication in Cultured Porcine Macrophages and Results in Significant Attenuation in Pigs

**DOI:** 10.3389/fmicb.2020.01123

**Published:** 2020-05-29

**Authors:** Lauro Velazquez-Salinas, Steven J. Pauszek, Lauren G. Holinka, Douglas P. Gladue, Steven I. Rekant, Elizabeth A. Bishop, Carolina Stenfeldt, Antonio Verdugo-Rodriguez, Manuel V. Borca, Jonathan Arzt, Luis L. Rodriguez

**Affiliations:** ^1^Foreign Animal Disease Research Unit, USDA/ARS Plum Island Animal Disease Center, Greenport, NY, United States; ^2^College of Veterinary Medicine and Animal Science, National Autonomous University of Mexico, Mexico City, Mexico; ^3^PIADC Research Participation Program, Oak Ridge Institute for Science and Education, Oak Ridge, TN, United States; ^4^Department of Veterinary Population Medicine, University of Minnesota, St. Paul, MN, United States

**Keywords:** vesicular stomatitis, pathogenesis, M51R, virulence, macrophages, immune response, type 1 interferon

## Abstract

In this study, we explore the virulence of vesicular stomatitis New Jersey virus (VSNJV) in pigs and its potential relationship with the virus’s ability to modulate innate responses. For this purpose, we developed a mutant of the highly virulent strain NJ0612NME6, containing a single amino acid substitution in the matrix protein (M51R). The M51R mutant of NJ0612NME6 was unable to suppress the transcription of genes associated with the innate immune response both in primary fetal porcine kidney cells and porcine primary macrophage cultures. Impaired viral growth was observed only in porcine macrophage cultures, indicating that the M51 residue is required for efficient replication of VSNJV in these cells. Furthermore, when inoculated in pigs by intradermal scarification of the snout, M51R infection was characterized by decreased clinical signs including reduced fever and development of less and smaller secondary vesicular lesions. Pigs infected with M51R had decreased levels of viral shedding and absence of RNAemia compared to the parental virus. The ability of the mutant virus to infect pigs by direct contact remained intact, indicating that the M51R mutation resulted in a partially attenuated phenotype capable of causing primary lesions and transmitting to sentinel pigs. Collectively, our results show a positive correlation between the ability of VSNJV to counteract the innate immune response in swine macrophage cultures and the level of virulence in pigs, a natural host of this virus. More studies are encouraged to evaluate the interaction of VSNJV with macrophages and other components of the immune response in pigs.

## Introduction

Vesicular stomatitis virus (VSV) is an arbovirus, and the prototype for the viral family Rhabdoviridae and the *Vesiculovirus* genus. Two distinct serotypes of VSV have been identified and defined: Indiana (VSIV) and New Jersey (VSNJV) (Rodriguez, 2002; Velazquez-Salinas et al., 2016b). VSNJV is responsible for most of the vesicular disease cases reported annually in the Americas (Rodriguez, 2002; Mead et al., 2009; Velazquez-Salinas et al., 2014). Because of the clinical resemblance between vesicular stomatitis (VS) and foot-and-mouth disease, one of the most economically devastating diseases for the livestock industry, quarantines and trade embargoes are often imposed in VS-affected premises (Timoney, 2016).

The ~11 kb RNA genome of VSV encodes five structural proteins: nucleocapsid (N), phosphoprotein (P), matrix (M), glycoprotein (G), and the large RNA-dependent RNA polymerase (L) (Dietzgen et al., 2011). The M protein is one of the best characterized proteins in the VSV proteome. Its functions are associated with apoptosis, virus assembly, budding, cytopathic effect, inhibition of host transcription, nucleocytoplasmic transport of host RNA, and inhibition of host interferon response (Ahmed et al., 2003; Rajani et al., 2012, Hastie et al., 2013; Melzer et al., 2017). In this context, the methionine residue at position 51 of the M protein plays a key role in maintaining optimal functionality of this protein. Substitution of this highly conserved residue for the amino acid arginine (M51R) results in profound loss of viral fitness by reducing the virus’s ability to block innate immune responses, causing increased production of type 1 interferon (IFN) and induction of interferon stimulated genes (Lyles et al., 1996; Stojdl et al., 2003; Ahmed et al., 2008; Varble et al., 2016; Melzer et al., 2017). Possible mechanisms associated with the dysfunctionality of M51R phenotype might involve its inability to inhibit the activity of host RNA polymerases (Ahmed et al., 2008) and its interaction with protein complexes like the nucleoporin Nup98, and the export RNA factor Rae1 (Faria et al., 2005; Rajani et al., 2012; Quan et al., 2014).

It is well-documented that VSNJV causes sporadic vesicular disease outbreaks in livestock in the southwestern U.S. at 8–10-year intervals. Each outbreak cycle is associated to a distinct viral strain among many circulating in endemic regions of Mexico (Rodriguez et al., 2000, Rainwater-Lovett et al., 2007, Arroyo et al., 2011; Velazquez-Salinas et al., 2014). However, the intrinsic and extrinsic factors associated with the emergence and spread of these particular strains remain unclear.

Recently, we described that an epidemic strain (NJ0612NME6), responsible for the most extensive VS outbreak in the southwestern US’s recent history, had an increased ability to disrupt innate immune responses in experimentally inoculated pigs, a natural host of this virus (Velazquez-Salinas et al., 2018a). Specifically, decreased levels of systemic type 1 IFN and tumor necrosis factor (TNF), along with increased levels of interleukin 6 were associated with more prolonged periods of higher fever, higher RNAemia, as well as an increased number of vesicular lesions in pigs infected with NJ0612NME6, when compared with a non-epidemic strain from southern Mexico (NJ0806VCB).

In this study, we aimed to gain further insight about the role of innate immune responses during the pathogenesis of VSNJV in pigs. For this purpose, we genetically engineered a cDNA clone of the highly virulent strain of VSNJV (NJ0612NME6) to introduce a single amino acid mutation in the M protein at position 51 to develop a mutant virus (M51R). For *in vitro* testing, we used both non-immune and immune cells. As non-immune cells, we used primary fetal porcine kidney cells that contain a mix of epithelial, fibroblasts, and endothelial-like cell phenotypes (Richter et al., 2012). Since skin and mucosal surfaces are considered to be the primary anatomical targets during the natural infection in livestock (Rodriguez et al., 2000), we considered this cellular mix appropriate to evaluate the innate immune response. As immune cells we used primary monocyte-derived macrophages. These cells have been successfully used to characterize the innate cellular response to infection by other viruses including African swine fever virus, classical swine fever, and VSV (Carlson et al., 2016; Velazquez-Salinas et al., 2016a, 2018b). Macrophages have been shown to play a preponderant role in the virulence of VSV in mice (Ciavarra et al., 2005; Junt et al., 2007; Simon et al., 2010). Additionally, we conducted a comprehensive *in-vivo* pathogenesis study using a previously described pig infection model (Martinez et al., 2003; Velazquez-Salinas et al., 2017, 2018b). The findings of this study are discussed in the context of the role that innate immune responses might play in determining the virulence of VSNJV in pigs.

## Materials and Methods

### Viral Strains

A high-titer virus stock (1 × 10^9.8^ TCID_50_/ml) of the highly virulent VSNJV NJ0612NME6 strain grown in baby hamster kidney cells (BHK-21) was used to construct a cDNA clone by site-direct recombination using a reverse genetics system as previously described (Velazquez-Salinas et al., 2018b, 2019).

### Cell Lines

Monkey kidney epithelial cells (Vero), BHK-21, Madin-Darby bovine kidney epithelial cells (MDBK-t/2), and primary fetal porcine kidney cell cultures (FPKC) were obtained from the Foreign Animal Disease Diagnostic Laboratory (FADDL) at the Plum Island Animal Disease Center (PIADC), Greenport, NY. Porcine monocytes-derived macrophages cell cultures (PM-MQC) were obtained as previously described (Zsak et al., 1996). The BSR-T7/5 cell line that constitutively expresses the T7 RNA polymerase (Buchholz et al., 1999) was used to recover the recombinant viruses.

For the purpose of this work, we considered stromal cells (epithelial and fibroblasts) like FPKC as non-immune cells, while that white blood cells that mediate innate immunity like PM-MQC where considered immune cells.

### Construction of the M51R Mutant Virus

LC-KAN-NJ0612NME6 plasmid containing the full-length genome of the highly virulent VSNJV NJ0612NME6 strain was used as a template to obtain the plasmid LC-KAN-NJ0612NME6-M51R carrying the codon substitution ATG-AGA at codon 51 of the matrix gene. This substitution was done using the QuickChange XL site-directed mutagenesis kit (Stratagene), following the manufacturer recommendations. For this purpose, the following set of primers was designed: M51R-F 5′-GATTTCTTCGGAAT GGAGGATAGAGACTTATATGACAAGGACTCCT-3′ and M51R-R 5′-AGGAGTCCTTGTCATATAAGTCTCTATCCTCCATTCCGAAGAAATC-3′.

To recover only the newly amplified plasmid NJ0612NME6-M51R, the site-directed mutagenesis reaction was digested with the type IIM restriction endonuclease Dpn1, and cloned into XL10-Gold ultra-competent cells.

### *In vitro* Rescue of the rNJ0612NME6 and M51R Viruses

Viral rescue was achieved from a cDNA VSV clone as previously described (Velazquez-Salinas et al., 2019). Briefly, independent transfections were conducted in BSR-T7/5 cells, using either LC-KAN-NJ0612NME6 or LC-KAN-NJ0612NME6-M51R plasmids and supporting plasmids pTIT-VSNJV-N, pTIT-VSNJV-P, and pTIT-VSNJV-L. After viral recovery, high titer viral stocks (HTVS) of rNJ0612NME6 (parental virus) and M51R viruses were made in Vero cells. Sequence identity of both viruses was confirmed by sequencing analysis as previously described (Pauszek and Rodriguez, 2012).

### Cell Viability Assay

To evaluate differences in the ability to induce cytotoxicity between rNJ0612NME6 and M51R viruses, we conducted the MTT assay. This assay is a rapid colorimetric technique for determining cellular growth and survival by assessing the enzymatic capability of the cell to reduce the tetrazolium dye MTT (Mosmann, 1983).

The MTT assay was conducted using the MTT Cell Proliferation Assay kit (ATCC bioproducts Cat#30-1010K) following the manufacturer instructions. Briefly, the MTT assay was conducted in 96-well plates using preformed monolayers of FPKC (~1 × 10^5^ cells per well). Wells were infected in octuplicate with 100 μl of either rNJ0612NME6 or M51R viruses at different MOI’s (10–0.00001). Twenty-four hours post-infection (hpi), 10 μl of MTT dye was added to each well and incubated for 2 h at 37°C. Finally, 100 μl of detergent reagent was added to each well and incubated for 2 h at room temperature. Plates were read in a microplate reader using an optical density of 570 nm. Optical values were converted to percentages of cell survival by comparing the values obtained from infected wells to the average value achieved by the negative control (DMEM). To confirm the ability of FPKC to mount an antiviral response, an additional set of plates was treated with 2 units/ml of recombinant porcine IFN-α2A per well 24 h prior to infection. Since the induction of apoptosis has been reported in different cell lines after treatment with IFN (Chawla-Sarkar et al., 2003), controls for this experiment included: FPKC treated only with IFN-α2A, and uninfected cells.

### *In vitro* Growth Characterization

To assess the ability of rNJ0612NME6 and M51R viruses to grow in FPKC and PM-MQC cells, multistep growth curves were performed in triplicate at a MOI of 0.01 TCID_50_. After 1 h of absorption (time zero), samples were collected at 1, 4, 8, 24, and 48 hpi. Viral titrations were conducted in BHK-21 cells by endpoint titration assay in 96-well plates as previously described (Velazquez-Salinas et al., 2018a). Final titers were determined by the Reed and Muench method and expressed as TCID_50_/ml (Reed and Muench, 1938).

### Transcriptional Immune Response

To evaluate the transcriptional immune profile associated with the *in vitro* infection of rNJ0612NME6 and M51R mutant viruses in FPKC and PM-MQC cells, a total of 42 pig genes were studied including: interferons type I (IFNα1, IFNα7-11, IFNα9, IFNα10, IFNα13, IFNα14, IFNα15, IFNα16, IFNα17, IFNβ1, IFNδ1, IFNδ2, IFNδ5-9-11, IFNδ6, IFNδ7, IFNδ8, IFNκ, IFNε, and IFNω1-6), type II (IFNγ) and type III (IFNλ1), interferon regulatory factors (IRF 1, IRF 3, IRF 6, IRF 7, and IRF 9), transcription factors (SAT 1 and SAT 2), interferon stimulated genes (ISG) (IFIT1, IFIT2, RIG-I, MX1, MX2, OAS1, GBP1, BST2, PKR, ISG20, and TRIM25), and chemokines and cytokines (IL-6, TNF-α, and CXCL10). Cells were infected at a MOI of 10 TCID_50_ and total cellular RNA was extracted after 5 hpi using the RNeasy Mini Kit (QIAGEN) following the manufacturer’s instructions. Analyses were conducted by quantitative reverse transcription real-time polymerase chain reaction (qRT-PCR) as previously described (Borca et al., 2008). A difference of at least threefold either up or down between normalized mRNA expression levels in VSV-infected cells and mock-infected cells was considered as significant (Brukman and Enquist, 2006; Borca et al., 2008).

### Plaque Assay

To evaluate the plaque morphology on FPKC produced by rNJ0612NME6 and M51R viruses, we conducted a standard plaque assay. Preformed monolayers of FPKC on six-well plates were infected with each virus, and incubated for 1 h at 37°C. Cells were then overlaid with tragacanth gum (0.6%) and incubated for 48 h at 37°C. Finally, cells were stained with crystal violet to make evident the plaque formation in infected cells.

### Animal Experiments

To compare the virulence and transmissibility of rNJ0612NME6 and M51R, two experiments (one for each virus) were conducted in pigs, a natural host of VSV, as previously described (Martinez et al., 2003; Velazquez-Salinas et al., 2017, 2018a). For each experiment, eight male, 8–10 weeks old Yorkshire pigs (~30 kg weight) were randomly segregated into two groups of four (directly inoculated and contact) and separated by double fencing to prevent direct contact. After 1 week of acclimation, pigs in the inoculated group were sedated using a mixture of xylazine, ketamine, and Telazol (4, 8, and 3 mg/kg, respectively) and infected by intradermal inoculation by scarification in the snout with a dose of 10^7^ TCID_50_ of virus in 50 μl of either rNJ0612NME6 or M51R. Twenty-four hours after infection, inoculated pigs were allowed to co-mingle with pigs from their respective contact group until the end of the experiment (day 21).

### Ethics Statement

Animal experiments were performed under biosafety level 3AG conditions in the animal facilities at PIADC. All experimental procedures were carried out in compliance with the Animal Welfare Act (AWA), the 2011 Guide for Care and Use of Laboratory Animals, the 2002 PHS Policy for the Humane Care and Use of Laboratory Animals, and U.S. Government Principles for Utilization and Care of Vertebrate Animals Used in Testing, Research, and Training (U.S. Office of Science and Technology Policy, 1985), as well as specific animal protocols reviewed and approved by the PIADC Institutional Animal Care and Use Committee of the US Departments of Agriculture and Homeland Security (protocol number #245-05-14R).

### Clinical Evaluation and Sampling

Clinical evaluation and sample collection were conducted daily in all animals from 0 to 10 days and at 14 and 21 days post-infection (dpi). Clinical evaluation included measurement of rectal temperatures and assessment of vesicular lesion development using a cumulative clinical scoring system based on the number and location of lesions as previously described (Velazquez-Salinas et al., 2018a). Briefly, maximum scores of 45 and 46 were allowed for direct inoculated and contact pigs, respectively. A characteristic lesion in each of the 16 digits contributed to the cumulative score with two points, while one or two points are assigned for lesions on the snout of directly inoculated and contact animals correspondingly, and two points added for lesions in carpal/tarsal skin, oral cavity, and lower lip. Sample collection included: whole blood, serum, oropharyngeal (OP) swabs, and nasal swabs. After collection, samples were processed as previously described (Velazquez-Salinas et al., 2018a) and stored at −70°C until testing.

### Post-mortem Sample Collection

To compare the biodistribution between rNJ0612NME6 and M51R, the two pigs displaying the highest clinical scores from each of the two groups (inoculated and contact) from each of the two experiments were chosen for tissue harvest at 21 dpi. Pigs were deeply sedated using an intramuscular injection of Telazol, ketamine, and xylazine at 4.5, 12, and 6 mg/kg, respectively, and posteriorly were humanely euthanized by exsanguination. Tissue collection included: tonsil of the soft palate, submandibular lymph node, spleen, snout skin, popliteal lymph node, and skeletal muscle. Duplicate aliquots of each sample containing 30 mg of each tissue were prepared and frozen at −70°C until processing.

### Viral RNA Detection

To contrast the viral growth dynamics during infection in pigs between rNJ0612NME6 and M51R, the presence of viral RNA extracted from different biological samples collected during the time course of the experiment was assessed by real-time RT-PCR (rRT-PCR). RNA extraction was conducted using the Ambion’s MagMax-96 Viral RNA Isolation Kit (Ambion, Austin, TX) as previously described (Arzt et al., 2010). rRT-PCR targeting the VSNJV nucleocapsid gene (N) was conducted as previously described (Scherer et al., 2007) but using a different forward primer (5′-GCACTTCCTGATGGGAAATCA-3′) to match the native sequence of the NJ0612NME6 viral strain (Velazquez-Salinas et al., 2018b). rRT-PCR was carried out using 2.5 μl of RNA on an ABI 7500 system (Applied Biosystems, Austin, TX). Results were expressed as RNA genome copy numbers per 2.5 μl of RNA.

### Virus Isolation

The detection of infectious virus in biological samples collected during animal experiments was conducted in BHK-21 cells. To eliminate potential bacterial contamination, samples were filtered through 0.45 μm Spin-X filter columns (Costar cat. No 8163). Afterwards, samples were diluted 1:5 in cell culture media and 500 μl of each dilution was used to overlay preformed BHK-21 cell monolayers in 24 well-plates. After 1 h of incubation at 37°C, 2 ml of maintenance media was added to each well and monitored for cytopathic effect (CPE) for 72 h. Positive samples were confirmed by rRT-PCR and titrated by endpoint titration assay in 96 well-plates as described in the previous sections.

### Detection of Systemic Type I IFN in Pigs

To contrast the systemic type I IFN induction between rNJ0612NME6 and M51R during experimental infection in pigs, serum samples collected during the acute stage of the infection were assessed by Mx-CAT reporter assay (Fray et al., 2001; Francois et al., 2005). Analyses were conducted on MDBK-t/2 cells using a CAT ELISA kit (Roche Applied Sciences, Indianapolis IN) as previously described (Perez-Martin et al., 2012, Fernandez-Sainz et al., 2015). A standard curve was created using recombinant IFN-α2A at concentrations from 1.95 to 1,000 U/ml and used to determine international units of antiviral activity per ml for each sample. The presence of type1 IFN in serum samples is positively correlated to the induction of the Mx gene in MDBK-t/2. This correlation has been previously validated (Francois et al., 2005), and the Mx-CAT reporter assay has been successfully used in previous experiments to analyze the presence of type I IFN in pigs (Fernandez-Sainz et al., 2015; Velazquez-Salinas et al., 2018a).

### Serum Neutralization Assay

The development of neutralizing antibodies against VSNJV during the infection of pigs with either rNJ0612NME6 or M51R was monitored by serum neutralization assay in Vero cells (96-well plates containing 1 × 10^6^ cells per plate), and using 1,000 TCID_50_ of VSNJV as previously described (Flanagan et al., 2001). Serum neutralizing activity was reported as the reciprocal of the highest dilution giving 100% inhibition of CPE.

### Statistical Analysis

Statistical significance between groups was determined using the Holm-Sidak method, with alpha = 0.05. Calculations were performed using GraphPad Prism version 8.01 for Windows (GraphPad Software, La Jolla, California, USA, www.graphpad.com).

## Results

**Figure 1:**
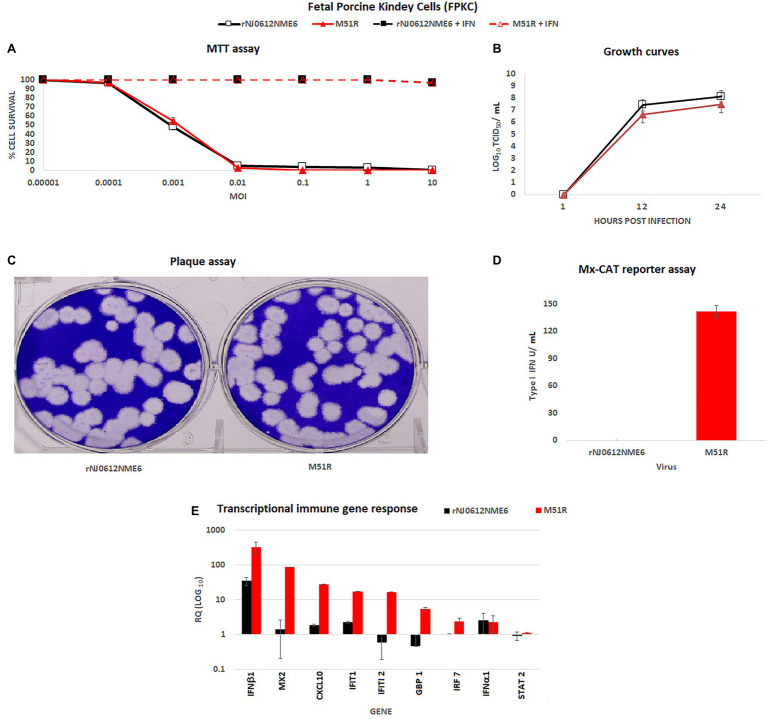
*In vitro* characterization of rNJ0612NME6 and M51R viruses in fetal porcine kidney cells (FPKC). **(A)** MTT assay was used to assess differences between both viruses to induce cytotoxicity in FPKC at different MOI’s, using untreated and previously treated cells with an external source of type I interferon (IFN) (2 units/ml of recombinant porcine IFN-α2A per well). Apoptosis was not detected in both controls: FPKC only treated with IFN-α2A, and untreated ones. **(B)** Multistep growth curves were performed in FPKC to compare virus yields between both viruses at specific times post-infection, using at initial MOI = 0.01. **(C)** Conventional plaque assay was conducted on FPKC to monitor differences in plaque formation between both viruses. **(D)** Mx-CAT reporter assay was used to test the ability of both viruses to induce the production of type I INF in FPKC. Samples were collected from multistep growth curves experiments at 24 hours post-infection (hpi). **(E)** Differences in the transcriptional immune gene response induced by different viruses on FPKC, were assessed by qRT-PCR. RQ values represent relative quantities of mRNA accumulation (estimated by 2^−ΔΔCt^) with their corresponding SD. All experiments were conducted in triplicates.

To gain a better understanding of the role of innate immune responses during the replication of VSNJV, FPKC (non-immune cells) and PM-MQC (immune cells) were used. When evaluated in FPKC cells, no differences were observed between rNJ0612NME6 and M51R in viral capacity to induce cytotoxicity at different MOI (Figure 1A). Conversely, 100% cell survival was recorded during the infection with either virus at any MOI when cells were pre-treated with an external source of type I IFN, demonstrating the competence of FPKC in responding to type I IFN stimulation (Figure 1A). Although M51R grew to slightly lower titer than rNJ0612NME6, no significant differences in the viral growth kinetics were found between viruses (Figure 1B).

Overall, both viruses produced similar plaque size and morphology (Figure 1C). However, a strong type I IFN response was detected only in M51R-infected cells (Figure 1D). The increased innate response in M51R infected cells was further confirmed by transcriptional analysis performed at 5 hpi. M51R induced significantly higher mRNA levels of IFNβ1, Mx2, CXCL10, IFT1, IFIT2 GBP1, and IRF7 compared to cells infected with rNJ0612NME6 (Figure 1E). Also, no significant differences were observed in the transcriptional levels of IFNα1 and STAT2 indicating that they were not induced by either of the two viruses.

**Figure 2:**
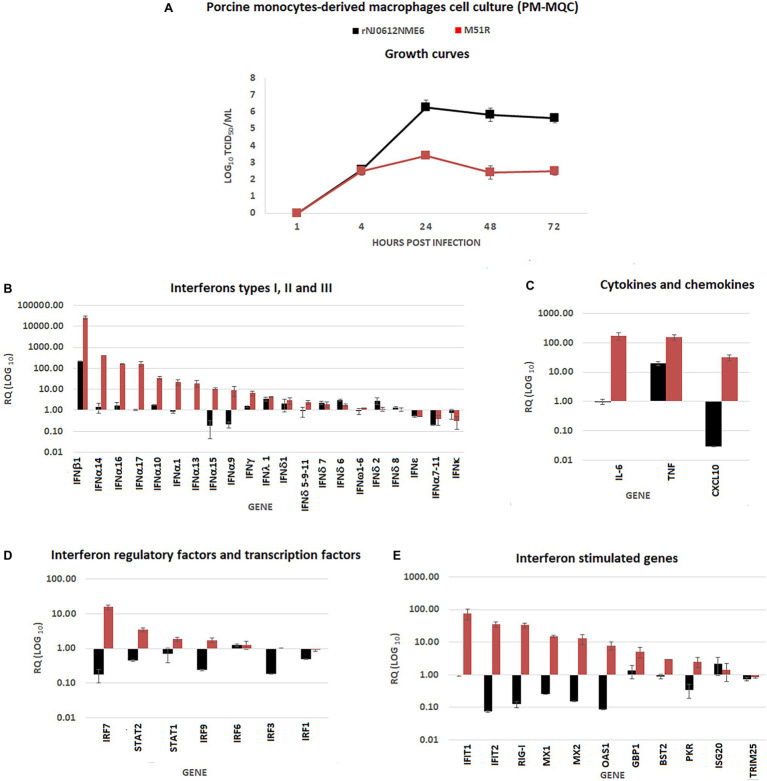
*In vitro* characterization of rNJ0612NME6 and M51R viruses in porcine monocyte-derived macrophages cell cultures (PM-MQC). **(A)** Multistep growth curves were performed in PM-MQC to compare virus yields between both viruses at specific times post-infection, using at initial MOI = 0.01. Differences in the transcriptional immune gene response induced by different viruses on PM-MQC, were assessed by qRT-PCR. RQ values represent relative quantities of mRNA accumulation (estimated by 2^−ΔΔCt^) with their corresponding SD in **(B)** type I, II and III IFN, **(C)** cytokines and chemokines, **(D)** Interferon regulatory factors, and transcription factors, and **(E)** Interferon stimulated genes. All experiments were conducted in triplicates.

In contrast, infection of PM-MQC using an MOI 0.01 revealed a significantly decreased capacity of M51R (~3 log reduction; *p* < 0.05) to grow in PM-MQC when compared with rNJ0612NME6 virus (Figure 2A). To analyze the transcriptional immune response, PM-MQC were infected with each virus at a MOI of 10 and samples were collected at 5 hpi and evaluated by qRT-PCR (Figures 2B–E). Infection with M51R resulted in a significant (*p* < 0.05) increase of mRNAs associated with type 1 IFNs including IFNβ1 and different subtypes of IFNα, as well as type II IFN (IFNγ). Conversely, rNJO0612NM6 was able to significantly (*p* < 0.05) decrease the transcriptional levels of IFNα9 and IFNα15 genes. No differences were observed in the transcriptional levels of type II IFN and other subtypes of type I IFN genes (Figure 2B).

Compared with rNJ0612NME6, infection with M51R resulted in a significant (*p* < 0.05) increase in mRNA for several cytokines and chemokines (i.e., IL-6, TNF, and CXCL10) (Figure 2C), interferon regulatory factors and transcription factors (IRF7, IRF9, STAT1, and STAT2) (Figure 2D), and ISG (IFIT1, IFIT2, MX1, MX2, OAS1, GBP1, BST2, and PKR) (Figure 2E).

### Virulence in Pigs

We compared the virulence between rNJ0612NME6 and M51R using a previously established model to assess the pathogenesis of VSV in pigs. The experimental design allowed not only evaluation of clinical signs (fever and vesicular lesions), viral shedding, RNAemia, and antibody response in directly inoculated and contact infected animals, but also assessed the ability of each virus to be transmitted by direct contact.

### Clinical Assessment

**Figure 3:**
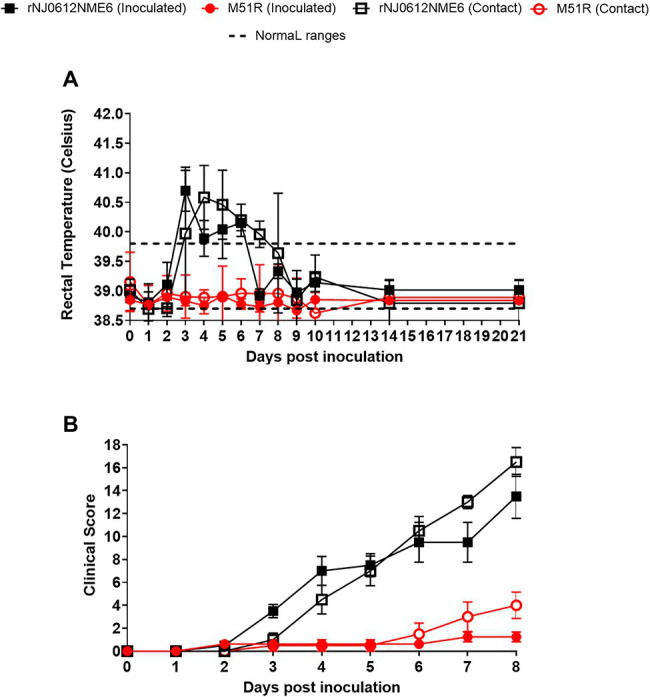
Clinical evaluation in pigs. Two independent groups of pigs were inoculated with 1 × 10^7^ TCID_50_ with either rNJ0612NME6 or M51R viruses. **(A)** Changes in rectal temperatures, denoting fever in pigs direct inoculated or infected by contact exposure within each group, were monitored daily during the experiments. Dashed lines represent the standard rectal temperature of an uninfected pig. **(B)** The development of vesicular lesions in snout, mouth, lips, feet was examined daily in both groups of pigs. In both groups, maximum clinical scores were reached at 8 dpi. In both cases values represent means and standard deviations of cumulative clinical scores within each group of pigs.

Rectal temperatures ≥39.8°C occurred in all pigs directly inoculated and contact-infected with rNJ0612NME6 at 3–7 dpi or days post exposure (dpe) (Figure 3A). Conversely, none of the pigs directly inoculated with M51R or those in direct contact had fever throughout the course of the experiment.

The development of vesicular lesions was monitored daily using the scoring system described above. Significantly higher clinical scores (*p* < 0.05) were observed in pigs infected with rNJ0612NME6 (directly inoculated: 13.5±1.9 and contact exposure: 16.5±1.2) than pigs infected with M51R (directly inoculated: 1.2 ± 0.4 and contact exposure: 4.0 ± 1.1) (Figure 3B).

**Figure 4:**
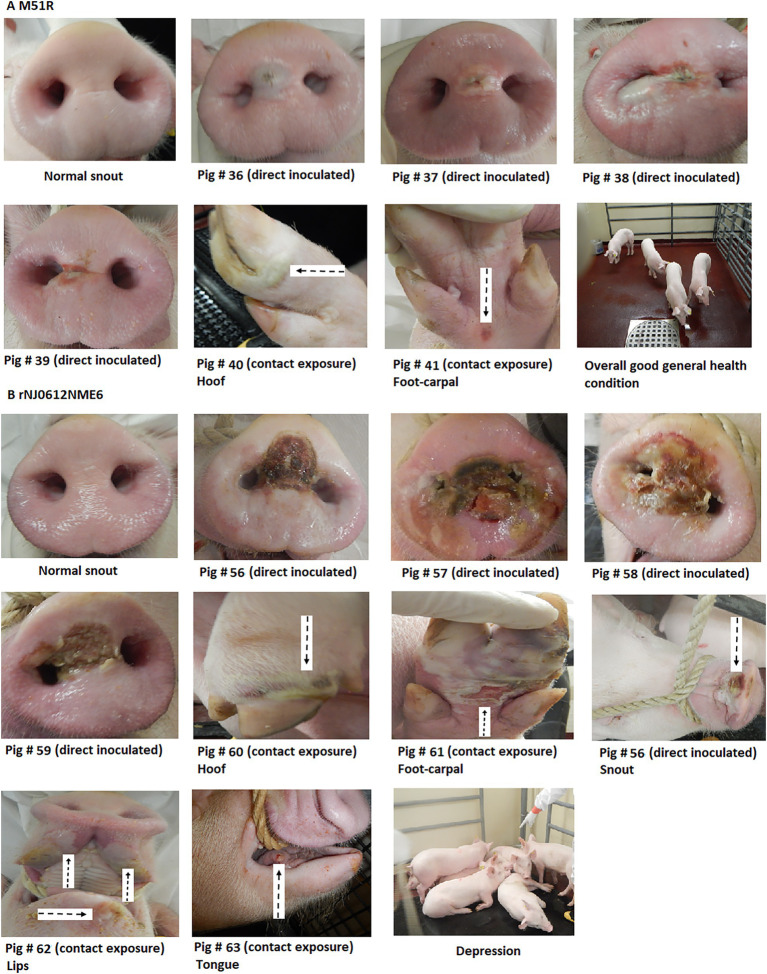
Clinical differences between groups of pigs infected with either rNJ0612NME6 or M51R viruses. **(A)** Despite the intrinsic characteristics of M51R virus to overcome the host innate immune response, clinical outcomes from different pigs infected within this group were characterized by the development of vesicular FIGURE 4lesions in both direct inoculated and contact exposure pigs. Overall pigs in this group kept a general health condition during the entire experiment (absence of fever, general depression, and lameness). **(B)** Contrasting differences were observed in pigs infected with rNJ0612NME6 in terms of vesicular lesion size development, systemic dissemination characterized for the development of vesicular lesions at different anatomic sites, and overall alteration of the health condition, especially during the acute phase of the infection.

Direct inoculation with M51R resulted in the development of small vesicular lesions at the site of inoculation with the exception of pig # 38, where the lesion extended outside the inoculation site (Figure 4A). In this animal, vesicular lesions developed at 2 dpi increased in size and ruptured between 3 and 5 dpi. No secondary lesions were observed, except in one animal, where two small vesicles were detected in the carpal region of the front limbs at 7 dpi. Although M51R was transmitted by contact exposure, vesicular lesions were small and appeared only in the carpal region of the back limbs of two of the four exposed pigs between 6 and 7 dpe (Figure 4A). These small lesions were confirmed positive for VSV by rRT-PCR (not shown).

No signs of obtundation or lameness were observed in any of the M51R infected animals throughout the course of the experiment.

Conversely, direct inoculation of pigs with rNJ0612NME6 resulted in the appearance of vesicular lesions at the inoculation sites at 2 dpi. Lesions increased in size considerably and ruptured around 3 and 5 dpi (Figure 4B). Vesicular lesions appeared at secondary sites starting at 3 dpi. All contact exposed animals developed lesions on the snout, lips, tongue, and feet (coronary bands, tarsal, and carpal regions) by 3 dpe (Figure 4B). The extensive size of the vesicular lesions on the feet of these animals was associated with severe lameness after 5 dpi or dpe and severe obtundation in all animals during the febrile period.

### Viral Shedding

The extent and duration of viral shedding in pigs was evaluated by the collection of daily nasal and oral swabs which were analyzed by rRT-PCR and viral isolation.

**Figure 5:**
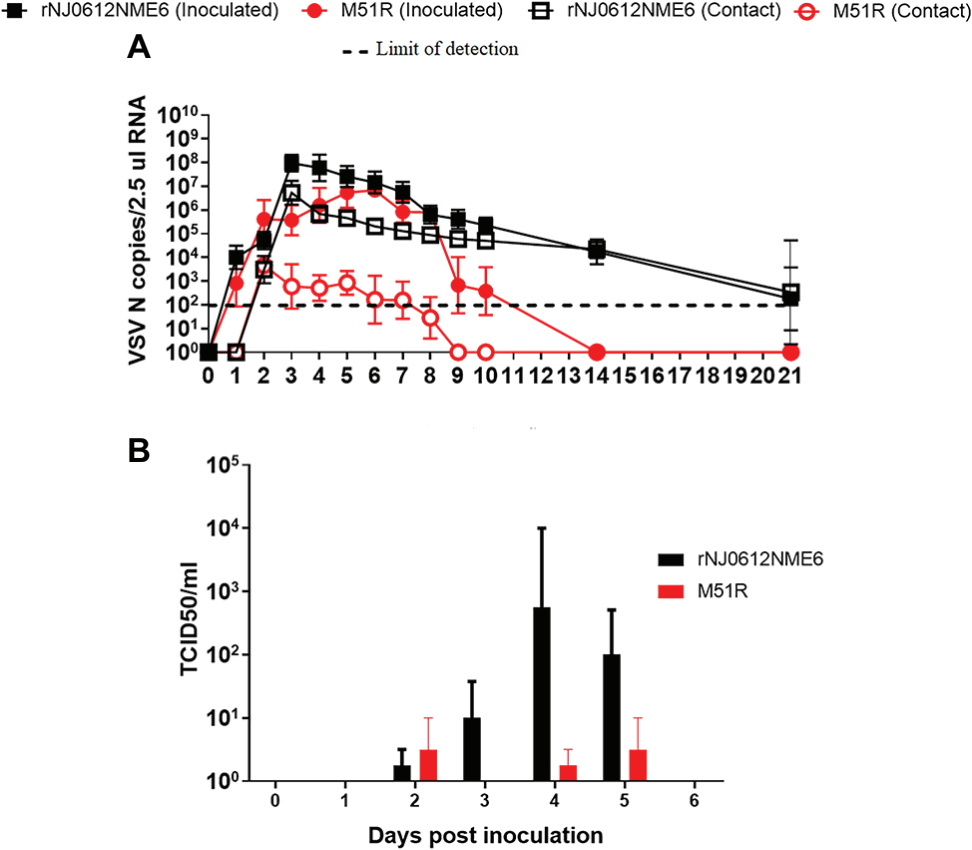
Differences of viral shedding on nasal swabs between groups of pigs infected with either rNJ0612NME6 or M51R viruses. **(A)** Levels of Vesicular stomatitis virus (VSV) nucleocapsid (N) RNA in different samples were quantified by rRT-PCR. Limit of detection of qRT-PCR using for this study was previously determined to be about 1.98 VSV N copies/2.5 μl RNA. **(B)** Viral isolations were conducted on Vero cells, and then viral titers from positive samples were determined and expressed in TCID_50_/ml. Values represent means and standard deviations within different groups of pigs.

There were significant differences (*p* < 0.05) between the two viruses both in the extent and duration of nasal shedding measured by rRT-PCR with rNJ0612NME6 shedding at higher levels and longer periods than M51R. While levels of viral RNA peaked by 3 dpi in pigs inoculated with rNJ0612NME6 and viral RNA was still detectable at 21 dpi, viral RNA peaked at 5 dpi in M51R inoculated animals and fell below level of detection by 14 dpi. These differences were more profound in contact-infected groups (Figure 5A). Similarly, infectious virus was recovered at higher titers and for longer periods in rNJ0612NME6 directly inoculated and contact animals. In M51R-infected animals, virus was recovered at lower titers and only from directly inoculated animals (Figure 5B). Virus was not recovered in nasal swabs of M51R contact exposed pigs. The peak of virus recovery in both groups was consistent with the rupture of the vesicles in the snout (Figure 5B).

**Figure 6:**
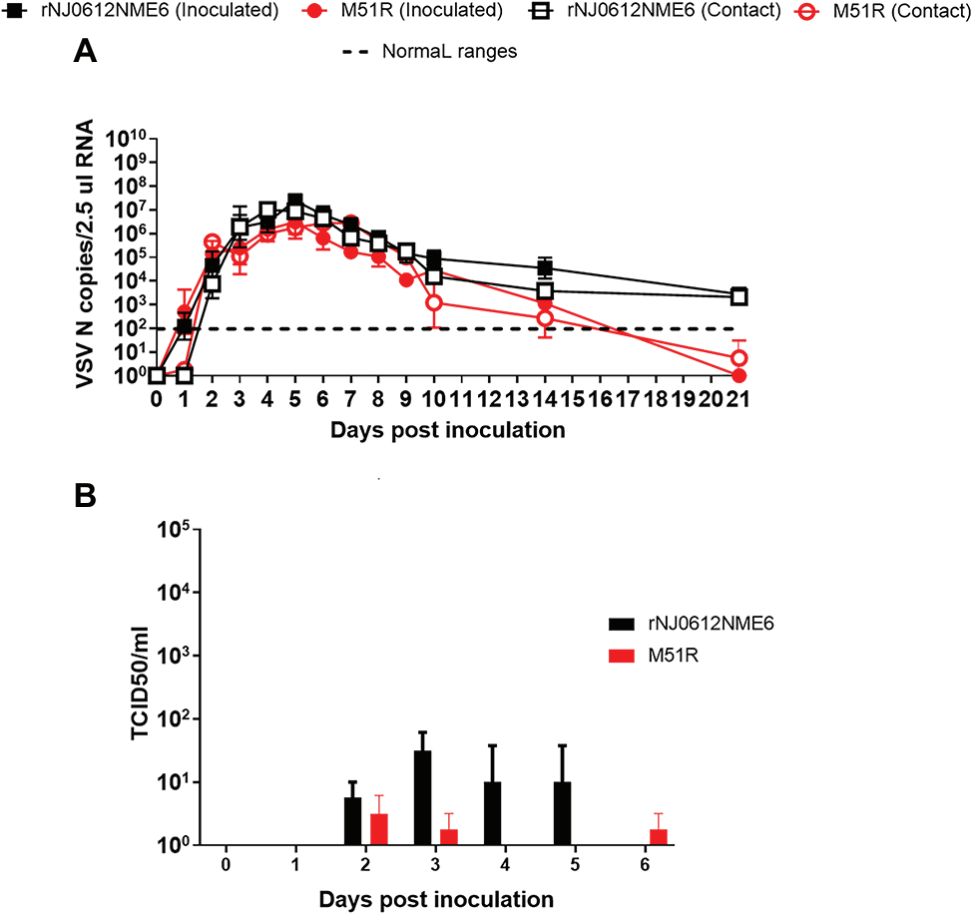
Differences of viral shedding on oral swabs between groups of pigs infected with either rNJ0612NME6 or M51R viruses. **(A)** Levels of VSV nucleocapsid (N) RNA in different samples were quantified by rRT-PCR. Limit of detection of qRT-PCR using for this study was previously determined to be about 1.98 VSV N copies/2.5 μl RNA. **(B)** Viral isolations were conducted on Vero cells, and then viral titers from positive samples were determined and expressed in TCID_50_/ml. Values represent means and standard deviations within different groups of pigs.

No significant differences in oral shedding of viral RNA were observed between groups of pigs infected with either of the two viruses. Viral RNA was detectable as early as 1 dpi and 1 dpe in directly inoculated or contact exposed pigs, respectively. Oral shedding peaked by 4 dpe and 5 dpi and decreased steadily for both viruses until day 14. By 21 dpi (20 dpe), only animals inoculated with rNJ0612NME6 had detectable viral RNA in oral swabs (Figure 6A). Infectious virus was detected intermittently and in low titers in oral swabs collected from pigs infected with either virus. Higher average titers of infectious virus were recovered from pigs infected with rNJ0612NME6 than with M51R between 2 and 6 dpi regardless the route of infection (Figure 6B).

### RNAemia

Although infectious VSV is not detectable in blood, the presence of RNAemia in experimentally infected pigs was first reported in a previous study as a potential marker for VSV virulence (Velazquez-Salinas et al., 2018a). The ability of rNJ0612NME6 and M51R to induce RNAemia (presence of viral RNA in blood) was assessed by rRT-PCR in whole blood samples collected during the course of the experiment.

**Figure 7:**
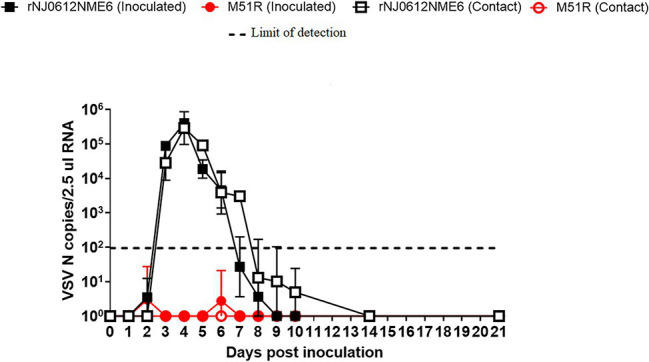
Differences of RNAemia levels between groups of pigs infected with either rNJ0612NME6 or M51R viruses. Levels of RNAemia were quantified by rRT-PCR from blood samples collected daily from all pigs during the course of this experiment. Limit of detection of qRT-PCR using for this study was previously determined to be about 1.98 VSV N copies/2.5μl RNA. Values represent means and standard deviations within different groups of pigs.

Consistent with its inability to induce severe clinical signs in pigs, animals infected with M51R did not have detectable RNAemia regardless of the route of infection (Figure 7). In contrast, regardless of the route of infection, rNJ0612NME6 infection resulted in severe clinical signs and a marked phase of RNAemia during the acute stage of infection. Levels of viral RNA in the blood of these pigs peaked at 4 dpi or 3 dpe and dropped below levels of detection by 7 dpi or dpe (Figure 7).

Consistent with previous findings (Velazquez-Salinas et al., 2018a), despite high levels of RNAemia in pigs infected with rNJ0612NME6, attempts to isolate infectious virus were unsuccessful despite using different cell lines (BHK-21 and Vero cells) and dilution of blood samples to mitigate the action of potential inhibiting factors.

### Systemic Type I IFN Response

**Figure 8:**
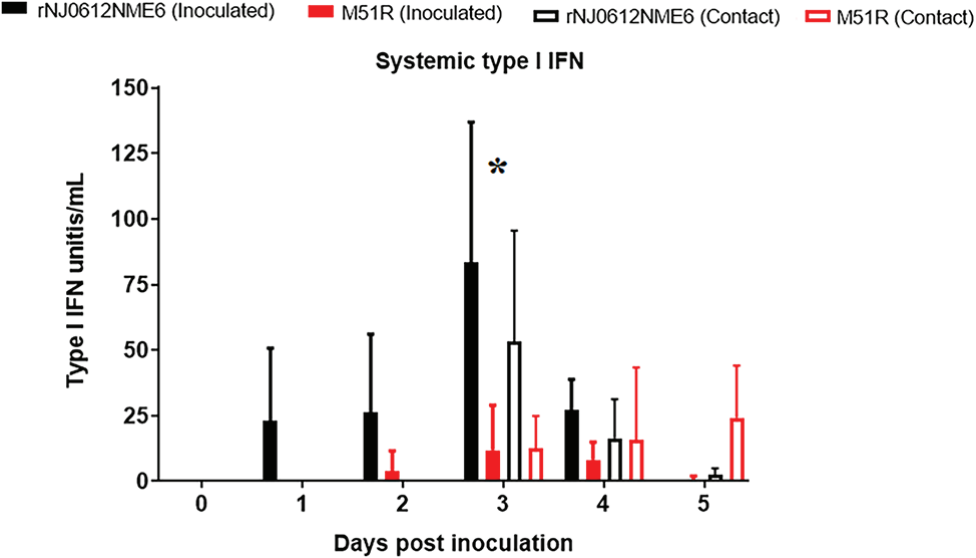
Differences in systemic levels of type I IFN induction between groups of pigs infected with either rNJ0612NME6 or M51R viruses. Mx-CAT reporter assay was used to monitor the systemic type I IFN production in pigs during the acute phase of the infection with different viruses. Values represent means and standard deviations within different groups of pigs. Asterisk at 3 dpi shows significant differences (*p* < 0.05) between pigs infected with rNJ0612NME6 and M51R viruses.

To evaluate induction of systemic type I IFN response in groups of pigs after infection with the different viruses, serum samples collected during the acute phase were evaluated by Mx-CAT-ELISA. In contrast to results in primary cells, where M51R was able to induce higher amounts of type I IFN than the parental virus, pigs infected with rNJ0612NME6 developed a faster and higher systemic type I IFN response than pigs infected with M51R (Figure 8).

Comparison between groups of pigs infected by the same route with different viruses showed statistically significant (*p* < 0.05) differences in IFN levels at 3 dpi. In M51R contact exposed pigs, IFN response was delayed, reflecting the delay in the development of vesicular lesions observed in this group of pigs.

### Adaptive Immune Response

**Figure 9:**
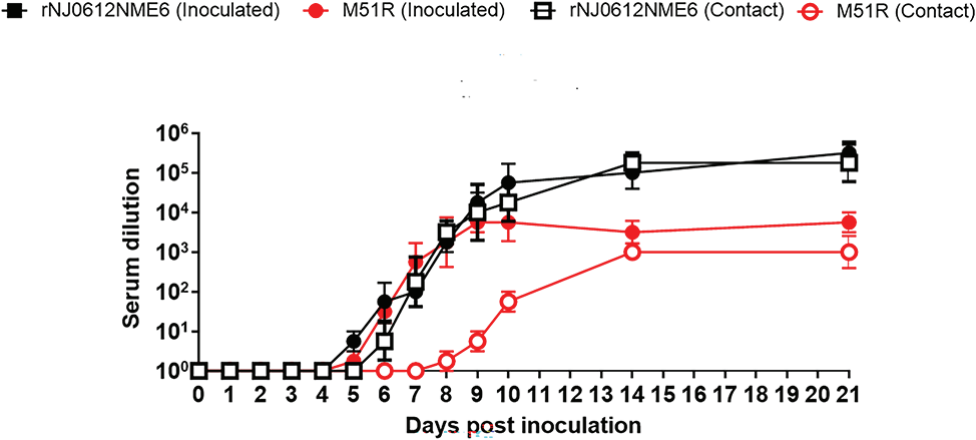
Differences in neutralizing antibody production between groups of pigs infected with either rNJ0612NME6 or M51R viruses. Viral neutralization assay was used to monitor the development of neutralizing antibody titers in pigs, using serum samples collected during the course of this experiment. Values represent means and standard deviations within different groups of pigs.

To evaluate the adaptive immune response, neutralizing antibodies were quantified by serum neutralization assay. Regardless of the virus, directly inoculated pigs developed similar antibody responses starting at 6 dpi. However, by 14 and 21 dpi, pigs inoculated with rNJ0612NME6 had significantly higher antibody levels (*p* < 0.05) than those inoculated with M51R (Figure 9).

Neutralizing antibody response in pigs infected by contact exposure with rNJ0612NME6 was detectable at 6 dpe, while this response was detectable around 9 dpe in pigs infected with M51R. By the end of the experiment, the level of neutralizing antibodies was ~100 fold higher in pigs infected by contact with rNJ0612NME6 than in those infected with M51R (Figure 9).

### Virus Detection in Tissues

**Figure 10:**
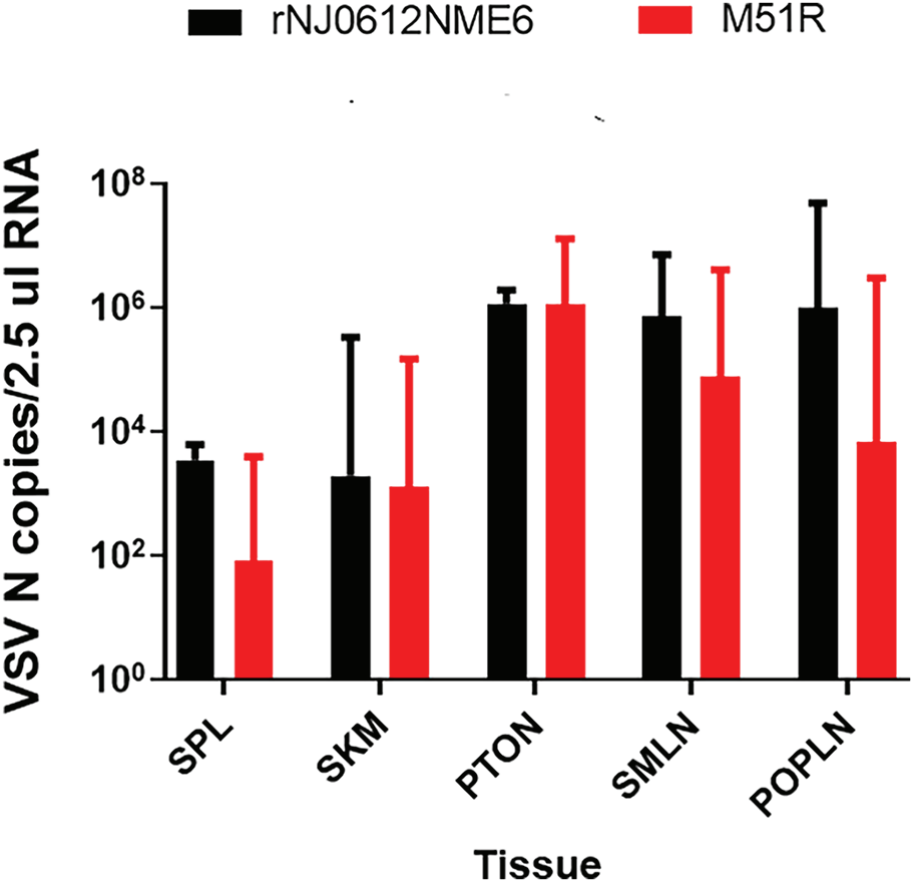
Differences in viral RNA accumulation in postmortem tissues between groups of pigs infected with either rNJ0612NME6 or M51R viruses. Postmortem tissues were collected at 21 dpi, and analyzed by qRT-PCR to detect VSV nucleocapsid RNA. Limit of detection of qRT-PCR using for this study was previously determined to be about 1.98 VSV N copies/2.5μl RNA. Values represent means and standard deviations within different groups of pigs. Abbreviations: tonsil of the soft palate (PTON), submandibular lymph node (SMLN), spleen (SPL), snout skin (SNT), popliteal lymph node (PopLN), and skeletal muscle (SKM).

Post mortem tissue collection was done at 21 dpi or dpe from the two pigs from each group with the highest clinical scores. Remarkably, despite their contrasting difference in virulence, no statistically significant difference was seen in the amount of viral RNA detected in the different tissues from rNJ0612NME6 and M51R infected pigs (Figure 10). Increased amounts of viral RNA was found in lymphoid tissues associated with anatomic sites of local replication including the tonsil of the soft palate, submandibular lymph node, and popliteal lymph node. Although not statistically significant, higher level of viral RNA was observed in the spleen collected from pigs infected with rNJ0612NME6 than in those infected by M51R, consistent with the increased capacity of rNJ0612NME6 virus to systemically disseminate during infection in pigs. Interestingly, despite the absence of RNAemia in the group of pigs infected with M51R virus, similar levels of viral RNA were found in skeletal muscle collected from pigs in both groups, suggesting that muscle tissue might play a role during the VSV infection in pigs (Figure 10). Finally, no infectious virus was recovered from any of the post mortem tissues collected during this study.

## Discussion

In general, very little is known about virus-host interactions during the infection of VSNJV in natural hosts. Understanding these processes is key to assessing intrinsic viral factors determining virulence in domestic animals. In a previous study, we reported that the increased virulence of the epidemic VSNJV strain NJ0612NME6, compared with its genetically closest endemic relative, might be associated with the ability of the former virus strain to modulate the innate immune responses in pigs (Velazquez-Salinas et al., 2018a). In this study, we introduced into NJ0612NME6 the M51R mutation shown to affect the viral ability to overcome innate responses (Ahmed et al., 2003; Rajani et al., 2012, Hastie et al., 2013; Melzer et al., 2017), to gain more insights about the role of innate responses during VSV infection in pigs. The outcomes of this study demonstrated that there is a positive correlation between the ability of VSNJV to replicate and overcome the innate immune responses in cultured macrophages and virulence in pigs.

*In vitro* studies were carried out in two types of primary cells: FPKC (non-immune cells) and PM-MQC (immune cells), representing different cellular environments that might be encountered by the virus during infection in pigs. Surprisingly, no significant differences in growth kinetics, viral yield, and plaque formation were seen between rNJ0612NME6 and M51R in FPKC despite significant differential transcription of ISG genes and the production of increased amounts of type I IFN observed in M51R-infected FPKC.

On the other hand, the M51R mutant had impaired growth kinetics in PM-MQC, indicating the essential role played by this residue in the replication of VSNJV in macrophages. The difference between FPKC and PM-MQC supporting replication of M51R may be explained by the intrinsic immune characteristics of each cell type and suggests that immune cells may play a critical role in VSNJV pathogenesis in pigs. While fibroblasts and epithelial cells are predominately producers of IFNβ (Ivashkiv and Donlin, 2014), macrophages express different pattern recognition receptors, and the constitutive expression of ISG like IRF-7 (a regulator of multiple of type I IFN), that allow these cells to mount a rapid antiviral response, reacting to initial picomolar concentrations of type I IFNs (Ning et al., 2011; Lazear et al., 2013; Ivashkiv and Donlin, 2014). This is consistent with the differences in the transcriptional profiles found between PM-MQC and FPKC after infection with rNJ0612NME6 and M51R, where the transcriptional profile of IRF-7 and IFN α in FPKC was not different between the two viruses.

Several studies have shown the relevant role that type I IFNs play as antiviral cytokines against VSV infection (Muller et al., 1994; Stojdl et al., 2003; Lichty et al., 2004). However, our results suggest that the effectiveness of type I IFNs against VSNJV in epithelial cells might vary depending on the source of IFN, whether it is external or internally induced. On one hand, our results showed the competence of FPKC to mount an effective antiviral state against the infection of either rNJ0612NME6 or M51R by prior stimulation with an external source of type I IFN. This is consistent with a previous report, showing that the oncolytic virus VSV-IFNβ-NIS coding for the human IFNβ (Naik and Russell, 2009) had an attenuated phenotype in FPKC and *in-vivo*, failing to produce epithelial lesions on the skin of infected pigs (Velazquez-Salinas et al., 2017).

On the other hand, we showed the inability of virus-induced type I IFN to fully contain an infection with M51R virus not only in FPKC, but remarkably also *in vivo*, as demonstrated by the formation of epithelial lesions (albeit small) in the snout of pigs direct inoculated with this virus. This suggests that epithelial cells of the skin might be less competent to mount an adequate antiviral response against VSNJV under natural infection. On this perspective, it has been reported that during natural infections in livestock, local epithelial lesions (without generalization) occur even in animals with significant levels of neutralizing antibodies induced by prior infection, suggesting that local processes might mediate lesion development (Rodriguez et al., 1990).

We have now shown that the M51R substitution in the matrix protein of VSNJV has a profound impact on the *in vivo* virulence of this virus, compromising its ability to systemically disseminate in infected pigs and thus reducing the formation and size of secondary vesicular lesions and the presentation of clinical signs including fever and obtundation. Interestingly, the correlation between the reduction in the capacity of M51R virus to replicate *in vitro* in macrophage cultures and the impairment to systemically disseminate *in vivo* is consistent with previous studies that show the essential role macrophages play to clear VSV during infection in mice by preventing systemic dissemination, supporting cytokine production, and facilitating the initiation of immune humoral responses (Ciavarra et al., 2005, 2006; Junt et al., 2007).

Furthermore, the clinical outcome in pigs infected with M51R resembles that shown by pigs experimentally infected with an attenuated VSIV strain, where clinical infection is characterized by the formation of vesicular lesions at the site of inoculation but the formation of secondary vesicular lesions and the presentation of fever are rarely observed (Martinez et al., 2003; Stallknecht et al., 2004). This suggests that there might be important differences between different strains in their ability to overcome the innate immune response in pigs. It has been suggested that interferon induction (or suppression) is a *quasi-species* marker in VSV. This is supported by prior observations that VSNJV field isolates derived from individual local lesions vary in their ability to repress induction of type I IFN (Marcus et al., 1998).

In addition, we detected the presence of viral RNA in pig tissues collected from both groups at 21 dpi, but we were not able to isolate infectious virus from any of those samples. This is consistent with previous studies in mice (Simon et al., 2010), cattle (Letchworth et al., 1996), and hamsters (Barrera and Letchworth, 1996), where the presence of viral RNA but no viral mRNA was detected. No infectious virus was detected at this stage of the disease indicating that VSV genomic or subgenomic RNA can persist in tissues from convalescent animals for long periods of up to 5 months (Letchworth et al., 1996). Future studies should be conducted to understand the acute infection dynamics and mechanisms of early VSV infection in pigs leading to the persistence of viral RNA (Stenfeldt et al., 2014). The M51R mutant virus could be a useful tool in studies to understand the role of different tissues and immune responses in VSV infection of pigs.

Finally, our results showed that the levels of systemic type I IFN were not associated with clinical signs in pigs infected with rNJ0612NME6 or M51R. These results contrast previous results comparing epidemic and endemic strains of VSNJV (Velazquez-Salinas et al., 2018a), where decreased type I IFN was seen in animals with severe disease. Our results showed that increased levels of type I IFN induced during infection are not always associated with protection in pigs and in some cases might act as a marker of disease at least at the local epithelium level. More studies are necessary to evaluate the role of innate immune responses mounted by different immune cell types involved in immune responses to infection with VSNJV.

Importantly, the contrasting clinical differences between pigs infected with rNJ0612NME6 or M51R confirm the relevance of RNAemia, fever, and intensity of antibody immune response as appropriate biological markers of virulence during the characterization of VSV strains in pigs. In the case of RNAemia, disparate clinical scores between groups of pigs infected with different viruses suggest that epithelial lesions are associated with RNAemia.

Also, despite its inability to fully inhibit innate responses, M51R was still capable of producing vesicular local lesions at inoculated sites, suggesting that other factors are relevant during local replication of VSNJV, whereas attenuated VSIV cause reduced or no lesions at the inoculation site (Martinez et al., 2003, 2004).

## Conclusion

Collectively, our results show that a highly virulent VSNJV containing a single amino acid mutation (M51R) is significantly attenuated in pigs and has impaired growth in pig immune cells, which is likely associated to increase type I IFN responses. The mutant virus retains its ability to grow in epithelial cells and can replicate locally in inoculated sites in pigs, suggesting different mechanisms of pathogenesis for local and systemic VSNJV infections. Furthermore, our results show the relevance of the M51R mutant as a model to better understand the immune interactions between VSNJV and other relevant natural hosts such as cattle and horses (Rodriguez, 2002). Based on previous studies, different clinical outcomes might be expected between pigs and cattle and horses during the infection with M51R (Howerth et al., 2006; Scherer et al., 2007; Mead et al., 2009).

## Data Availability Statement

The datasets generated for this study are available on request to the corresponding author.

## Ethics Statement

The animal study was reviewed and approved by the PIADC Institutional Animal Care and Use Committee of the US Departments of Agriculture and Homeland Security (protocol number #245-05-14R).

## Author Contributions

LV-S, SP, AV-R, and LR conceived and designed the experiments. LV-S, SP, CS, LH, DG, SR, and EB performed the experiments. LV-S, SP, and LR analyzed the data. LR, MB, and JA contributed the reagents, materials, and analysis tools. LV-S, SP, CS, MB, LH, DG, EB, SR, AV-R, JA, and LR wrote the manuscript.

## Conflict of Interest

The authors declare that the research was conducted in the absence of any commercial or financial relationships that could be construed as a potential conflict of interest.

## References

[ref2] Ahmed M. Mckenzie M. O. Puckett S. Hojnacki M. Poliquin L. Lyles D. S. (2003). Ability of the matrix protein of vesicular stomatitis virus to suppress beta interferon gene expression is genetically correlated with the inhibition of host RNA and protein synthesis. J. Virol. 77, 4646–4657. 10.1128/JVI.77.8.4646-4657.2003, PMID: 12663771 PMC152115

[ref1] Ahmed M. Marino T. R. Puckett S. Kock N. D. Lyles D. S. (2008). Immune response in the absence of neurovirulence in mice infected with mice protein mutant vesicular stomatitis virus. J. Virol. 82, 9273–9277. 10.1128/JVI.00915-08, PMID: 18614644 PMC2546904

[ref3] Arroyo M. Perez A. M. Rodriguez L. L. (2011). Characterization of the temporal and spatial distribution and reproductive ratio of vesicular stomatitis outbreaks in Mexico in 2008. Am. J. Vet. Res. 72, 233–238. 10.2460/ajvr.72.2.233, PMID: 21281198

[ref77] Arzt J. Pacheco J. M. Rodriguez L. L. (2010). The early pathogenesis of foot-and-mouth disease in cattle after aerosol inoculation. Identification of the nasopharynx as the primary site of infection. Vet Pathol. 47, 1048–1063. 20587691 10.1177/0300985810372509

[ref4] Barrera J. C. Letchworth G. J. (1996). Persistence of vesicular stomatitis virus New Jersey RNA in convalescent hamsters. Virology 219, 453–464. 10.1006/viro.1996.0271, PMID: 8638411

[ref5] Borca M. V. Gudmundsdottir I. Fernandez-Sainz I. J. Holinka L. G. Risatti G. R. (2008). Patterns of cellular gene expression in swine macrophages infected with highly virulent classical swine fever virus strain Brescia. Virus Res. 138, 89–96. 10.1016/j.virusres.2008.08.009, PMID: 18796318

[ref6] Brukman A. Enquist L. W. (2006). Suppression of the interferon-mediated innate immune response by pseudorabies virus. J. Virol. 80, 6345–6356. 10.1128/JVI.00554-06, PMID: 16775323 PMC1488972

[ref7] Buchholz U. J. Finke S. Conzelmann K. K. (1999). Generation of bovine respiratory syncytial virus (BRSV) from cDNA: BRSV NS2 is not essential for virus replication in tissue culture, and the human RSV leader region acts as a functional BRSV genome promoter. J. Virol. 73, 251–259. 10.1128/JVI.73.1.251-259.1999, PMID: 9847328 PMC103829

[ref8] Carlson J. O’donnell V. Alfano M. Velazquez Salinas L. Holinka L. G. Krug P. W.. (2016). Association of the host immune response with protection using a live attenuated African swine fever virus model. Viruses 8:291. 10.3390/v8100291, PMID: 27782090 PMC5086623

[ref9] Chawla-Sarkar M. Lindner D. J. Liu Y. F. Williams B. R. Sen G. C. Silverman R. H.. (2003). Apoptosis and interferons: role of interferon-stimulated genes as mediators of apoptosis. Apoptosis 8, 237–249. 10.1023/A:1023668705040, PMID: 12766484

[ref10] Ciavarra R. P. Stephens A. Nagy S. Sekellick M. Steel C. (2006). Evaluation of immunological paradigms in a virus model: are dendritic cells critical for antiviral immunity and viral clearance? J. Immunol. 177, 492–500. 10.4049/jimmunol.177.1.492, PMID: 16785546

[ref11] Ciavarra R. P. Taylor L. Greene A. R. Yousefieh N. Horeth D. Van Rooijen N.. (2005). Impact of macrophage and dendritic cell subset elimination on antiviral immunity, viral clearance and production of type 1 interferon. Virology 342, 177–189. 10.1016/j.virol.2005.07.031, PMID: 16143360

[ref150] Dietzgen R. G. Calisher C. H. Kurath G. Kuzmin I. V. Rodriguez L. L. Stone D. M. (2012). Family Rhabdoviridae in “Virus Taxonomy”. eds. King A. M. Q. Adams M. J. Carstens E. B. Lefkowitz E. J. (Oxford: Elsevier Academic Press), 686–714.

[ref14] Faria P. A. Chakraborty P. Levay A. Barber G. N. Ezelle H. J. Enninga J.. (2005). VSV disrupts the Rae1/mrnp41 mRNA nuclear export pathway. Mol. Cell 17, 93–102. 10.1016/j.molcel.2004.11.023, PMID: 15629720

[ref15] Fernandez-Sainz I. Ramanathan P. O’donnell V. Diaz-San Segundo F. Velazquez-Salinas L. Sturza D. F.. (2015). Treatment with interferon-alpha delays disease in swine infected with a highly virulent CSFV strain. Virology 483, 284–290. 10.1016/j.virol.2015.04.024, PMID: 26004252

[ref16] Flanagan E. B. Zamparo J. M. Ball L. A. Rodriguez L. L. Wertz G. W. (2001). Rearrangement of the genes of vesicular stomatitis virus eliminates clinical disease in the natural host: new strategy for vaccine development. J. Virol. 75, 6107–6114. 10.1128/JVI.75.13.6107-6114.2001, PMID: 11390612 PMC114326

[ref17] Francois C. Bernard I. Castelain S. Charleston B. Fray M. D. Capiod J. C.. (2005). Quantification of different human alpha interferon subtypes and pegylated interferon activities by measuring MxA promoter activation. Antimicrob. Agents Chemother. 49, 3770–3775. 10.1128/AAC.49.9.3770-3775.2005, PMID: 16127052 PMC1195395

[ref18] Fray M. D. Mann G. E. Charleston B. (2001). Validation of an Mx/CAT reporter gene assay for the quantification of bovine type-I interferon. J. Immunol. Methods 249, 235–244. 10.1016/S0022-1759(00)00359-8, PMID: 11226480

[ref20] Hastie E. Cataldi M. Marriott I. Grdzelishvili V. Z. (2013). Understanding and altering cell tropism of vesicular stomatitis virus. Virus Res. 176, 16–32. 10.1016/j.virusres.2013.06.003, PMID: 23796410 PMC3865924

[ref21] Howerth E. W. Mead D. G. Mueller P. O. Duncan L. Murphy M. D. Stallknecht D. E. (2006). Experimental vesicular stomatitis virus infection in horses: effect of route of inoculation and virus serotype. Vet. Pathol. 43, 943–955. 10.1354/vp.43-6-943, PMID: 17099151

[ref22] Ivashkiv L. B. Donlin L. T. (2014). Regulation of type I interferon responses. Nat. Rev. Immunol. 14, 36–49. 10.1038/nri3581, PMID: 24362405 PMC4084561

[ref23] Junt T. Moseman E. A. Iannacone M. Massberg S. Lang P. A. Boes M.. (2007). Subcapsular sinus macrophages in lymph nodes clear lymph-borne viruses and present them to antiviral B cells. Nature 450, 110–114. 10.1038/nature06287, PMID: 17934446

[ref25] Lazear H. M. Lancaster A. Wilkins C. Suthar M. S. Huang A. Vick S. C.. (2013). IRF-3, IRF-5, and IRF-7 coordinately regulate the type I IFN response in myeloid dendritic cells downstream of MAVS signaling. PLoS Pathog. 9:e1003118. 10.1371/annotation/4de7ddfd-52df-4f87-8ca4-d48afe646ca8, PMID: 23300459 PMC3536698

[ref26] Letchworth G. J. Barrera J. C. Fishel J. R. Rodriguez L. (1996). Vesicular stomatitis New Jersey virus RNA persists in cattle following convalescence. Virology 219, 480–484. 10.1006/viro.1996.0275, PMID: 8638415

[ref27] Lichty B. D. Power A. T. Stojdl D. F. Bell J. C. (2004). Vesicular stomatitis virus: re-inventing the bullet. Trends Mol. Med. 10, 210–216. 10.1016/j.molmed.2004.03.003, PMID: 15121047

[ref28] Lyles D. S. Mckenzie M. O. Kaptur P. E. Grant K. W. Jerome W. G. (1996). Complementation of M gene mutants of vesicular stomatitis virus by plasmid-derived M protein converts spherical extracellular particles into native bullet shapes. Virology 217, 76–87. 10.1006/viro.1996.0095, PMID: 8599238

[ref29] Marcus P. I. Rodriguez L. L. Sekellick M. J. (1998). Interferon induction as a quasispecies marker of vesicular stomatitis virus populations. J. Virol. 72, 542–549. 10.1128/JVI.72.1.542-549.1998, PMID: 9420257 PMC109406

[ref30] Martinez I. Barrera J. C. Rodriguez L. L. Wertz G. W. (2004). Recombinant vesicular stomatitis (Indiana) virus expressing New Jersey and Indiana glycoproteins induces neutralizing antibodies to each serotype in swine, a natural host. Vaccine 22, 4035–4043. 10.1016/j.vaccine.2004.03.065, PMID: 15364454

[ref31] Martinez I. Rodriguez L. L. Jimenez C. Pauszek S. J. Wertz G. W. (2003). Vesicular stomatitis virus glycoprotein is a determinant of pathogenesis in swine, a natural host. J. Virol. 77, 8039–8047. 10.1128/JVI.77.14.8039-8047.2003, PMID: 12829843 PMC161932

[ref32] Mead D. G. Lovett K. R. Murphy M. D. Pauszek S. J. Smoliga G. Gray E. W.. (2009). Experimental transmission of vesicular stomatitis New Jersey virus from *Simulium vittatum* to cattle: clinical outcome is influenced by site of insect feeding. J. Med. Entomol. 46, 866–872. 10.1603/033.046.0419, PMID: 19645291

[ref33] Melzer M. K. Lopez-Martinez A. Altomonte J. (2017). Oncolytic vesicular stomatitis virus as a viro-immunotherapy: defeating cancer with a "hammer" and "anvil". Biomedicine 5:8. 10.3390/biomedicines5010008, PMID: 28536351 PMC5423493

[ref34] Mosmann T. (1983). Rapid colorimetric assay for cellular growth and survival: application to proliferation and cytotoxicity assays. J. Immunol. Methods 65, 55–63. 10.1016/0022-1759(83)90303-4, PMID: 6606682

[ref35] Muller U. Steinhoff U. Reis L. F. Hemmi S. Pavlovic J. Zinkernagel R. M.. (1994). Functional role of type I and type II interferons in antiviral defense. Science 264, 1918–1921. 10.1126/science.8009221, PMID: 8009221

[ref36] Naik S. Russell S. J. (2009). Engineering oncolytic viruses to exploit tumor specific defects in innate immune signaling pathways. Expert. Opin. Biol. Ther. 9, 1163–1176. 10.1517/14712590903170653, PMID: 19637971

[ref37] Ning S. Pagano J. S. Barber G. N. (2011). IRF7: activation, regulation, modification and function. Genes Immun. 12, 399–414. 10.1038/gene.2011.21, PMID: 21490621 PMC4437765

[ref38] Pauszek S. J. Rodriguez L. L. (2012). Full-length genome analysis of vesicular stomatitis New Jersey virus strains representing the phylogenetic and geographic diversity of the virus. Arch. Virol. 157, 2247–2251. 10.1007/s00705-012-1420-x, PMID: 22825698

[ref39] Perez-Martin E. Weiss M. Diaz-San Segundo F. Pacheco J. M. Arzt J. Grubman M. J.. (2012). Bovine type III interferon significantly delays and reduces the severity of foot-and-mouth disease in cattle. J. Virol. 86, 4477–4487. 10.1128/JVI.06683-11, PMID: 22301155 PMC3318609

[ref41] Quan B. Seo H. S. Blobel G. Ren Y. (2014). Vesiculoviral matrix (M) protein occupies nucleic acid binding site at nucleoporin pair (Rae1 * Nup98). Proc. Natl. Acad. Sci. U. S. A. 111, 9127–9132. 10.1073/pnas.1409076111, 24927547 PMC4078809

[ref42] Rainwater-Lovett K. Pauszek S. J. Kelley W. N. Rodriguez L. L. (2007). Molecular epidemiology of vesicular stomatitis New Jersey virus from the 2004-2005 US outbreak indicates a common origin with Mexican strains. J. Gen. Virol. 88, 2042–2051. 10.1099/vir.0.82644-0, 17554039

[ref43] Rajani K. R. Pettit Kneller E. L. Mckenzie M. O. Horita D. A. Chou J. W. Lyles D. S. (2012). Complexes of vesicular stomatitis virus matrix protein with host Rae1 and Nup98 involved in inhibition of host transcription. PLoS Pathog. 8:e1002929. 10.1371/journal.ppat.1002929, PMID: 23028327 PMC3460625

[ref44] Reed L. J. Muench H. A. (1938). Simple method of estimating fifty percent endpoints. Am. J. Hyg. 27, 493–497.

[ref45] Richter A. Kurome M. Kessler B. Zakhartchenko V. Klymiuk N. Nagashima H.. (2012). Potential of primary kidney cells for somatic cell nuclear transfer mediated transgenesis in pig. BMC Biotechnol. 12:84. 10.1186/1472-6750-12-84, PMID: 23140586 PMC3537537

[ref47] Rodriguez L. L. (2002). Emergence and re-emergence of vesicular stomatitis in the United States. Virus Res. 85, 211–219. 10.1016/S0168-1702(02)00026-6, PMID: 12034487

[ref48] Rodriguez L. L. Bunch T. A. Fraire M. Llewellyn Z. N. (2000). Re-emergence of vesicular stomatitis in the western United States is associated with distinct viral genetic lineages. Virology 271, 171–181. 10.1006/viro.2000.0289, PMID: 10814582

[ref49] Rodriguez L. L. Vernon S. Morales A. I. Letchworth G. J. (1990). Serological monitoring of vesicular stomatitis New Jersey virus in enzootic regions of Costa Rica. Am. J. Trop. Med. Hyg. 42, 272–281. 10.4269/ajtmh.1990.42.272, PMID: 2156464

[ref50] Scherer C. F. O’donnell V. Golde W. T. Gregg D. Estes D. M. Rodriguez L. L. (2007). Vesicular stomatitis New Jersey virus (VSNJV) infects keratinocytes and is restricted to lesion sites and local lymph nodes in the bovine, a natural host. Vet. Res. 38, 375–390. 10.1051/vetres:2007001, PMID: 17506968

[ref51] Simon I. D. Van Rooijen N. Rose J. K. (2010). Vesicular stomatitis virus genomic RNA persists in vivo in the absence of viral replication. J. Virol. 84, 3280–3286. 10.1128/JVI.02052-09, PMID: 20032173 PMC2838132

[ref52] Stallknecht D. E. Greer J. B. Murphy M. D. Mead D. G. (2004). Effect of strain and serotype of vesicular stomatitis virus on viral shedding, vesicular lesion development, and contact transmission in pigs. Am. J. Vet. Res. 65, 1233–1239. 10.2460/ajvr.2004.65.1233, PMID: 15478770

[ref53] Stenfeldt C. Pacheco J. M. Rodriguez L. L. Arzt J. (2014). Early events in the pathogenesis of foot-and-mouth disease in pigs; identification of oropharyngeal tonsils as sites of primary and sustained viral replication. PLoS One 9:e106859. 10.1371/journal.pone.0106859, PMID: 25184288 PMC4153717

[ref54] Stojdl D. F. Lichty B. D. Tenoever B. R. Paterson J. M. Power A. T. Knowles S.. (2003). VSV strains with defects in their ability to shutdown innate immunity are potent systemic anti-cancer agents. Cancer Cell 4, 263–275. 10.1016/S1535-6108(03)00241-1, PMID: 14585354

[ref55] Timoney P. (2016). Vesicular stomatitis. Vet. Rec. 179, 119–120. 10.1136/vr.i4075, PMID: 27474058

[ref151] U.S. Office of Science and Technology Policy (1985). Laboratory animal welfare; U.S. government principles for the utilization and care of vertebrate animals used in testing, research and training; notice. Fed. Regist. 50, 20864–20865. PMID:11655791

[ref56] Varble A. J. Ried C. D. Hammond W. J. Marquis K. A. Woodruff M. C. Ferran M. C. (2016). The vesicular stomatitis virus matrix protein inhibits NF-kappaB activation in mouse L929 cells. Virology 499, 99–104. 10.1016/j.virol.2016.09.009, PMID: 27643886 PMC5102766

[ref57] Velazquez-Salinas L. Naik S. Pauszek S. J. Peng K. W. Russell S. J. Rodriguez L. L. (2017). Oncolytic recombinant vesicular stomatitis virus (VSV) is nonpathogenic and nontransmissible in pigs, a natural host of VSV. Hum. Gene. Ther. Clin. Dev. 28, 108–115. 10.1089/humc.2017.015, PMID: 28514874 PMC5583557

[ref58] Velazquez-Salinas L. Pauszek S. J. Barrera J. Clark B. A. Borca M. V. Verdugo-Rodriguez A.. (2019). Validation of a site-specific recombination cloning technique for the rapid development of a full-length cDNA clone of a virulent field strain of vesicular stomatitis New Jersey virus. J. Virol. Methods 265, 113–116. 10.1016/j.jviromet.2019.01.003, PMID: 30639413

[ref59] Velazquez-Salinas L. Pauszek S. J. Stenfeldt C. O’hearn E. S. Pacheco J. M. Borca M. V.. (2018a). Increased virulence of an epidemic strain of vesicular stomatitis virus is associated with interference of the innate response in pigs. Front. Microbiol. 9:1891. 10.3389/fmicb.2018.01891, PMID: 30158915 PMC6104175

[ref60] Velazquez-Salinas L. Pauszek S. J. Verdugo-Rodriguez A. Rodriguez L. L. (2018b). Complete genome sequences of two vesicular stomatitis New Jersey viruses representing the 2012 U.S. epidemic strain and its closest relative endemic strain from southern Mexico. Genome Announc. 6:e00049-18. 10.1128/genomeA.00049-18, PMID: 29449388 PMC5814489

[ref61] Velazquez-Salinas L. Pauszek S. J. Zarate S. Basurto-Alcantara F. J. Verdugo-Rodriguez A. Perez A. M.. (2014). Phylogeographic characteristics of vesicular stomatitis New Jersey viruses circulating in Mexico from 2005 to 2011 and their relationship to epidemics in the United States. Virology 449, 17–24. 10.1016/j.virol.2013.10.025, PMID: 24418533

[ref62] Velazquez-Salinas L. Risatti G. R. Holinka L. G. O’donnell V. Carlson J. Alfano M.. (2016a). Recoding structural glycoprotein E2 in classical swine fever virus (CSFV) produces complete virus attenuation in swine and protects infected animals against disease. Virology 494, 178–189. 10.1016/j.virol.2016.04.007, PMID: 27110709

[ref63] Velazquez-Salinas L. Zarate S. Eschbaumer M. Pereira Lobo F. Gladue D. P. Arzt J.. (2016b). Selective factors associated with the evolution of codon usage in natural populations of arboviruses. PLoS One 11:e0159943. 10.1371/journal.pone.0159943, PMID: 27455096 PMC4959722

[ref65] Zsak L. Lu Z. Kutish G. F. Neilan J. G. Rock D. L. (1996). An African swine fever virus virulence-associated gene NL-S with similarity to the herpes simplex virus ICP34.5 gene. J. Virol. 70, 8865–8871. 10.1128/JVI.70.12.8865-8871.1996, PMID: 8971015 PMC190983

